# Detoxifying Enzymes at the Cross-Roads of Inflammation, Oxidative Stress, and Drug Hypersensitivity: Role of Glutathione Transferase P1-1 and Aldose Reductase

**DOI:** 10.3389/fphar.2016.00237

**Published:** 2016-08-04

**Authors:** Francisco J. Sánchez-Gómez, Beatriz Díez-Dacal, Elena García-Martín, José A. G. Agúndez, María A. Pajares, Dolores Pérez-Sala

**Affiliations:** ^1^Department of Chemical and Physical Biology, Centro de Investigaciones Biológicas, Consejo Superior de Investigaciones CientíficasMadrid, Spain; ^2^Department of Pharmacology, University of ExtremaduraCáceres, Spain; ^3^Instituto de Investigaciones Biomédicas Alberto Sols (Consejo Superior de Investigaciones Científicas-Universidad Autónoma de Madrid), and Grupo de Hepatología Molecular, Instituto de Investigación Sanitaria del Hospital Universitario La Paz (IdiPAZ)Madrid, Spain

**Keywords:** glutathione transferase, aldose reductase, inflammation, oxidative stress, detoxification, allergy, drug adduct, drug hypersensitivity

## Abstract

Phase I and II enzymes are involved in the metabolism of endogenous reactive compounds as well as xenobiotics, including toxicants and drugs. Genotyping studies have established several drug metabolizing enzymes as markers for risk of drug hypersensitivity. However, other candidates are emerging that are involved in drug metabolism but also in the generation of danger or costimulatory signals. Enzymes such as aldo-keto reductases (AKR) and glutathione transferases (GST) metabolize prostaglandins and reactive aldehydes with proinflammatory activity, as well as drugs and/or their reactive metabolites. In addition, their metabolic activity can have important consequences for the cellular redox status, and impacts the inflammatory response as well as the balance of inflammatory mediators, which can modulate epigenetic factors and cooperate or interfere with drug-adduct formation. These enzymes are, in turn, targets for covalent modification and regulation by oxidative stress, inflammatory mediators, and drugs. Therefore, they constitute a platform for a complex set of interactions involving drug metabolism, protein haptenation, modulation of the inflammatory response, and/or generation of danger signals with implications in drug hypersensitivity reactions. Moreover, increasing evidence supports their involvement in allergic processes. Here, we will focus on GSTP1-1 and aldose reductase (AKR1B1) and provide a perspective for their involvement in drug hypersensitivity.

## Introduction

Drug hypersensitivity reactions pose an important clinical problem. They reduce the therapeutic armamentarium and may entail great severity, being life threatening in some cases. These reactions are mediated by the activation of the immune system by drugs or their metabolites. This can occur through the direct interaction of the drug/metabolite with receptors from immune cells or by covalent attachment of the drug to endogenous proteins, in a process known as haptenation. It is often considered that drugs are too small structures to activate the immune system on their own, whereas haptenated proteins or peptides can fulfill this role and be processed and presented by antigen presenting cells. In addition, factors leading to the exacerbation of the inflammatory response, the generation of danger signals or oxidative stress, contribute to the development of hypersensitivity reactions through mechanisms not completely understood.

Detoxifying and metabolic enzymes play multiple roles in cell homeostasis and may participate in drug hypersensitivity through various mechanisms. Metabolites produced by drug transformation carried out by these enzymes could activate the immune system. In addition, detoxifying enzymes play important roles in the control of inflammation, cellular redox status, and cytotoxicity.

Inflammation and oxidative stress cooperate in the pathogenesis of allergic diseases. A situation of oxidative stress may concur with sensitization and favor Th2 responses (Utsch et al., 2015). Moreover, oxidative stress induction is common to chemical allergens, including those that induce type IV hypersensitivity (Corsini et al., 2013). Indeed, numerous drugs, including doxorubicin, dapsone, cisplatin, sulfamethoxazole, and many others, elicit oxidative stress through multiple mechanisms (Bhaiya et al., 2006; Deavall et al., 2012; Hargreaves et al., 2016), increasing the generation of danger signals that act as coactivators for the allergic reaction (Sanderson et al., 2006). In turn, oxidative stress can increase the formation of drug-protein adducts by favoring the generation of reactive metabolites of drugs, thus facilitating protein haptenation and subsequent activation of the immune system or other toxic effects. Furthermore, oxidized proteins may be more susceptible to the addition of certain drugs or drug metabolites (Lavergne et al., 2009). Oxidative stress can also alter the ratio between reduced and oxidized glutathione species by depletion of the reduced form (GSH), thus favoring protein glutathionylation and/or reducing the possibility of drug detoxification through GSH conjugation. Conversely, it has been reported that antioxidants such as N-acetylcysteine, ebselen, and pyrrolidine dithiocarbamate can ameliorate immune and allergic responses in several models (Matsue et al., 2003; Monick et al., 2003; Galbiati et al., 2011). Importantly, a reduced antioxidant or cytoprotective capacity has been evidenced in allergy and asthma (Lutter et al., 2015), and sensitization to certain allergens is associated with inadequate antioxidant responses. Consequently, it has been proposed that exploring the master regulator of antioxidant responses Nuclear factor erythroid 2-related factor 2 (Nrf-2), may provide novel biomarkers for determining the sensitization potential of several chemicals (Natsch and Emter, 2008; Ade et al., 2009).

Recently, we have studied two types of detoxifying enzymes, GST and AKR (Sánchez-Gómez et al., 2007, 2010; Díez-Dacal et al., 2016), which interact with several drugs and are important players in the regulation of inflammation and redox status. Indeed, genetic variations in these enzymes have been associated with an increased risk of suffering diseases with an important allergic component such as atopy or asthma. Nevertheless, whereas the role of other drug metabolizing enzymes, such as cytochromes, in drug hypersensitivity has been frequently explored (Gueant et al., 2008; Bhattacharyya et al., 2014), those of GST and AKR remain poorly understood. Here, we provide a perspective on the interactions of GSTP1-1 and AKR1B1 with both drugs and factors contributing to allergic reactions, and suggest avenues to assess their potential as drug hypersensitivity biomarkers.

## GSTP1-1

**Figure 1:**
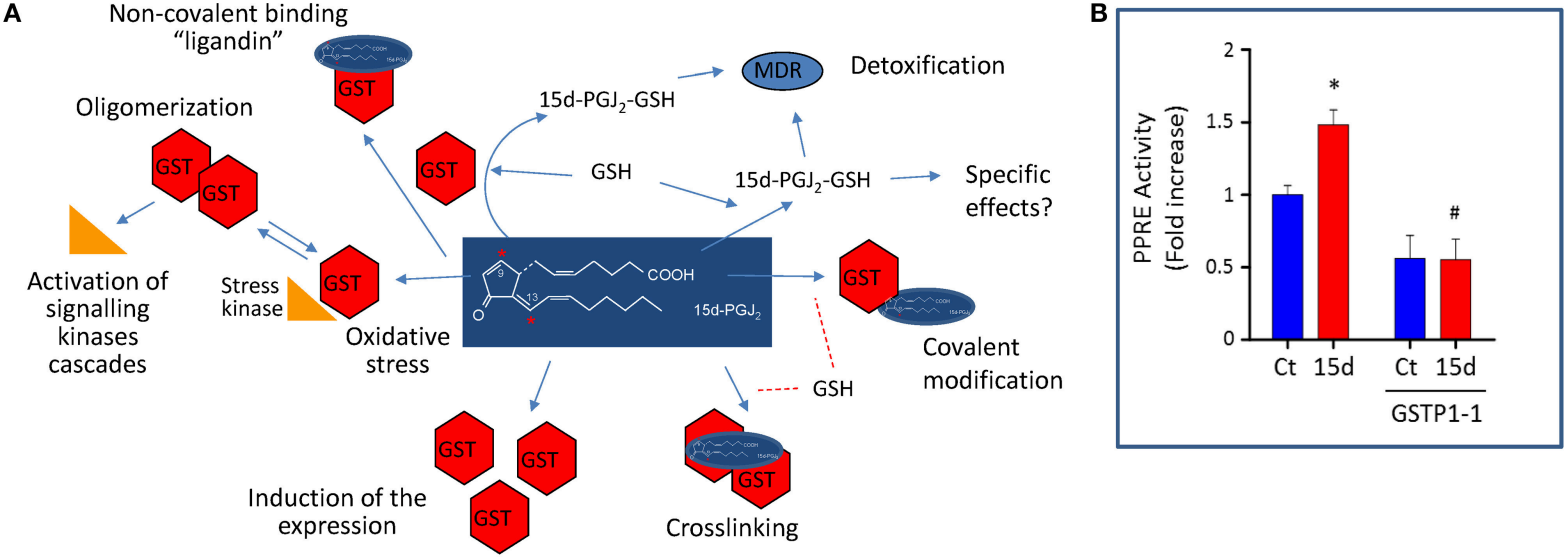
**Interactions of GSTP1-1 with the cyPG 15d-PGJ_2_ as a model of an electrophilic compound that can act both as a substrate and an inhibitor of the enzyme. (A)** GSTP1-1 (depicted as “GST” in the scheme) can detoxify electrophilic mediators, like 15d-PGJ_2_, and drugs, by conjugating them with GSH or through its ligandin role. In turn, GSTP1-1 can be covalently modified and/or cross linked by these compounds. Crosslinking or oligomerization secondary to drug-induced oxidative stress can impact stress signaling cascades. In addition, electrophilic drugs or mediators can induce GSTP1-1 expression in a cell-type dependent manner. **(B)** The ability of GSTP1-1 to detoxify and reduce the effects of 15d-PGJ_2_ is illustrated: GSTP1-1 overexpression blocks the activity of a PPAR promoter reporter element (PPRE) in cells. Rat mesangial cells were transfected with PPRE as previously described (Zorrilla et al., 2010), and with a GSTP1-1 expression vector where indicated. Then cells were treated in the absence (Ct) or presence of 15d-PGJ_2_ (15d) and the promoter activity measured by luminescence. The overexpression of GSTP1-1 was sufficient to block PPAR activation induced by the prostaglandin. ^*^p > 0.05 vs. Ct, ^#^*p* < 0.05 vs. 15d-PGJ_2_. Values represent mean ± SEM from three different experiments.

Glutathione-S-transferases are phase II enzymes that detoxify numerous endogenous and exogenous compounds by conjugation with GSH (Hayes et al., 2005). GSH-conjugates can then be exported from cells by the multidrug transporter system (Díez-Dacal and Pérez-Sala, 2012). Numerous genetic variations in GST enzymes have been identified and their functional consequences have been the subject of previous review (Board and Menon, 2013). Regarding GSTP1-1, the polymorphisms described have been mostly studied in the context of cancer and drug metabolism. However, in addition to its metabolic function, GSTP1-1 modulates stress response cascades by mechanisms involving protein-protein interactions with signaling proteins, like c-Jun terminal Kinase (JNK) and other mitogen activated protein kinases, Peroxiredoxin 6 (Prdx6), and Tumor necrosis factor (TNF)-associated factor 2 (TRAF2; Adler et al., 1999; Wu et al., 2006). Moreover, GSTP1-1 facilitates protein glutathionylation, thus regulating protein activity (Tew, 2007). Therefore, a complex landscape appears in which GSTP1-1 integrates cellular responses to redox stress by catalytic, protein-protein interaction and posttranslational mechanisms (Figure 1).

### Interaction of GSTP1-1 with oxidative stress

GSTP1-1 is a key factor for cellular adaptation to oxidative stress at multiple levels. *GSTP1-1* expression is strongly induced by oxidative stress as a defense mechanism through the binding of transcription factors, like Nrf-2 and activator protein (AP)-1, to the antioxidant response elements in its promoter (Kawamoto et al., 2000; Hayes et al., 2005). In turn, oxidative stress can reversibly inactivate GSTP1-1 by intramolecular disulfide formation or oligomerization (Shen et al., 1993; Sánchez-Gómez et al., 2010). Moreover, several electrophilic agents, including endogenous reactive mediators and drugs, induce an irreversible crosslinking of the enzyme (Sánchez-Gómez et al., 2013). The main residues involved in these modifications are the most reactive cysteines in GSTP1-1, namely, Cys47, and/or Cys101. Both, GSTP1-1 oligomerization and crosslinking affect its interactions with signaling proteins and stress cascades, as mentioned above.

GSTP1-1 can promote the reversible incorporation of GSH (S-glutathionylation) into low pKa cysteine residues of proteins. This modification modulates protein function, but also protects cysteine residues from further irreversible oxidations (Tew, 2007; Townsend et al., 2009), allowing the reduced form to be regenerated. Proteins S-glutathionylated by GSTP1-1 include Prdx6 (Manevich and Fisher, 2005), AKR1B1, and GSTP1-1 itself (Townsend et al., 2009; Wetzelberger et al., 2010).

Altogether, this evidence illustrates the complex redox regulation of GSTP1-1. Under mild oxidative stress, induction of GSTP1-1 expression and its redox “recycling” function afford cellular protection. However, pharmacological treatments or acute inflammation can inactivate GSTP1-1 either by direct oxidation and/or chemical inhibition. In both cases, allelic variants of GSTP1-1, namely, wild type GSTP1-1 (Ile105, Ala114) and variants: GSTP1-1(Ile105Val, Ala114), GSTP1-1(Ile105Val, Ala114Val), and GSTP1-1(Ile105, Ala114Val), differentially exert protective functions on protein activity and lipid peroxidation, which may influence susceptibility to oxidative stress of subjects carrying the various forms (Manevich et al., 2013).

### Interaction of GSTP1-1 with drugs

GSTP1-1 displays multiple interactions with drugs, either catalyzing their detoxification by GSH conjugation or being inactivated by them. These interactions are crucial for cancer therapy. GSTP1-1 overexpression is an important factor involved in tumor chemoresistance (Díez-Dacal and Pérez-Sala, 2012), and therefore, an important drug target, for which structurally diverse inhibitors, including ethacrynic acid, glutathione analogs, GSTP1-1 activatable drugs, and natural compounds have been considered (Singh, 2015). The mechanism of action of these compounds frequently involves binding to cysteine residues and/or GSTP1-1 oligomerization, as it occurs with electrophilic prostaglandins (PGs) or chlorambucil (Sánchez-Gómez et al., 2013). Interestingly, the pattern of GSTP1-1 crosslinking and/or chemical modifications depends on the presence of both substrates and inhibitors, for which this enzyme can be considered a converging platform for the effects of drugs and danger signals arising from oxidative stress or inflammation (Sánchez-Gómez et al., 2013).

GSTP1-1 also keeps important direct or indirect interactions with the mechanism of action of drugs such as acetaminophen (McGarry et al., 2015), acetylsalycilic acid (Baranczyk-Kuzma and Sawicki, 1997), and other non-steroidal anti-inflammatory drugs (Orhan and Sahin, 2001). In fact, GSTP1-1 deficiency correlates with higher acetaminophen toxicity in mice (McGarry et al., 2015). Also, a “ligandin” role of GSTP1-1 should be taken into account, since this abundant cytosolic enzyme can sequester drugs, thus reducing their effective concentrations (Oakley et al., 1999; Lu and Atkins, 2004).

### Interaction of GSTP1-1 with inflammatory mediators

GSTP1-1 also displays multiple interactions with inflammation: it is induced by proinflammatory stimuli, but this could exert a negative feedback on the inflammatory response. GSTP1-1 ameliorates the inflammatory response in several experimental models of tissue damage or inflammation (Xue et al., 2005; Luo et al., 2009). Interestingly, several GST, including GSTP1-1, attenuate the action of the inflammatory mediator 15-deoxy-Δ^12, 14^-PGJ_2_ (15d-PGJ_2_; Paumi et al., 2004). Evidence from our laboratory indicates that overexpression of GSTP1-1 in rat mesangial cells reduces the capacity of 15d-PGJ_2_ to activate Peroxisome proliferator activated receptor (PPAR) transcription factor(s) (Figure 1). Moreover, a reduction in the basal PPAR activity is also observed, suggesting the inactivation of endogenous PPAR agonists or the participation of additional mechanisms in GSTP1-1 regulation of inflammation.

In turn, electrophilic mediators like 15d-PGJ_2_ can inhibit GST activity in several cell types through various mechanisms (Sánchez-Gómez et al., 2007). Interestingly, cyclopentenone prostaglandins (cyPG) with dienone structure induce an extensive intermolecular crosslinking of GSTP1-1 monomers, involving mainly Cys47 and Cys101 (Sánchez-Gómez et al., 2013) that is blocked by GSH or non-metabolizable GSH analogs, indicating that cyPG-GSTP1-1 interaction is impaired in the GSH-bound enzyme.

Therefore, the interaction of GSTP1-1 with inflammatory mediators like cyPG is a two-way process strongly dependent on GSH availability (Gayarre et al., 2005; Díez-Dacal and Pérez-Sala, 2010), since the enzyme can conjugate electrophilic mediators with GSH, whereas cyPG can induce the expression and/or inhibit GST activity in a cell type-dependent manner (Sánchez-Gómez et al., 2007). Some of these interactions have also been evidenced for other GST isoforms (Gilot et al., 2002; Kudoh et al., 2014). These observations illustrate the intricate implications of GST in inflammation, with the net outcome depending on the delicate balance of all these factors.

### GSTP1-1 in allergic reactions

Although GST have been mostly studied in the fields of oxidative stress and chemoresistance, an interesting role in allergic reactions is emerging. Endogenous GSTP1-1 is an important target for haptenation, which has been related to the induction of certain drug hypersensitivity reactions (Meng et al., 2014). In addition, genetic variants of several GST isoforms have been found to associate with allergic processes including asthma (Tamer et al., 2004), drug eruptions (Ates et al., 2004), sensitization to thimerosal (Westphal et al., 2000), or allergic rhinitis (Iorio et al., 2014). In the case of GSTP1-1, both down- and up-regulations of GSTP1-1 levels have been reported in association with asthma (Schroer et al., 2011): whereas low levels could contribute to asthma, oxidative stress associated with the allergic response could induce GSTP1-1 expression. These changes in expression may in turn be modulated by the occurrence of polymorphisms, like Ile105Val (rs 1695; Dragovic et al., 2014), since this variant has been reported to display a reduced ability to conjugate several electrophilic drugs and reactive metabolites to GSH, and may associate with certain allergic diseases, including atopy and asthma (Hoskins et al., 2013). Polymorphic forms of GSTP1-1 correlate with the aggravation of asthma symptoms induced by air pollution (Su et al., 2013), and increased risk of asthma associated with acetaminophen (Kang et al., 2013) and exercise (Islam et al., 2009). In addition, the Ile105 wild type enzyme associates with enhancement of certain nasal allergic responses (Gilliland et al., 2004), whereas, according to another study, the Ala114 wild type enzyme associates with increased risk of atopy (Schultz et al., 2010). Nevertheless, lack of association of GSTP1-1 polymorphisms with allergic diseases or drug hypersensitivity has been reported in other studies, potentially due to differences in the genetic backgrounds of the patient cohorts studied.

Altogether, these findings support the role of GSTP1-1 as a risk factor in hypersensitivity responses by multiple mechanisms, given its multifunctional involvement in drug metabolism and inflammation. Moreover, GSTP1-1 emerges as a key factor to be considered in future genomic studies related with allergy development and drug hypersensitivity reactions.

## AKR1B1

AKR1B1 (or aldose reductase) is a member of the AKR superfamily, which comprises multiple enzymes involved in oxidoreduction of endogenous and exogenous compounds, including aliphatic and aromatic aldehydes, monosaccharides, steroids, aromatic hydrocarbons (PAHs), or isoflavonoids, using NADH or NADPH as cofactors. Structurally, this phase I metabolizing enzyme (Penning and Drury, 2007) is folded into a (α/β)_8_-barrel motif that is highly conserved among the members of this family and harbors the active site at its C-terminal end (Jez et al., 1997).

AKR1B1 primary role is to afford constitutive and inducible protection against toxic aldehydes generated under oxidative stress (Jin and Penning, 2007; Lyon et al., 2013). AKR1B1 reduces highly reactive lipid peroxidation products like 4-hydroxy-2-nonenal (HNE), acrolein, and methylglyoxal, as well as GSH-conjugates of these aldehydes such as glutathionyl-4-hydroxy-2-nonenal (GS-HNE) and GS-acrolein (Kolb et al., 1994; Srivastava et al., 1998; Vander Jagt et al., 2001). For instance, AKR1B1 activation played a cardioprotective role in rat myocardial ischemia by decreasing the accumulation of lipid peroxidation products in the ischemic heart (Kaiserova et al., 2008). Similarly, induction of AKR1B1 expression in response to oxidative stress plays a role in the antioxidant response (Wang et al., 2012). AKR1B1 also participates in steroid hormones catabolism and plays an important role in the regulation of steroid function in several tissues (Barski et al., 2008).

**Figure 2:**
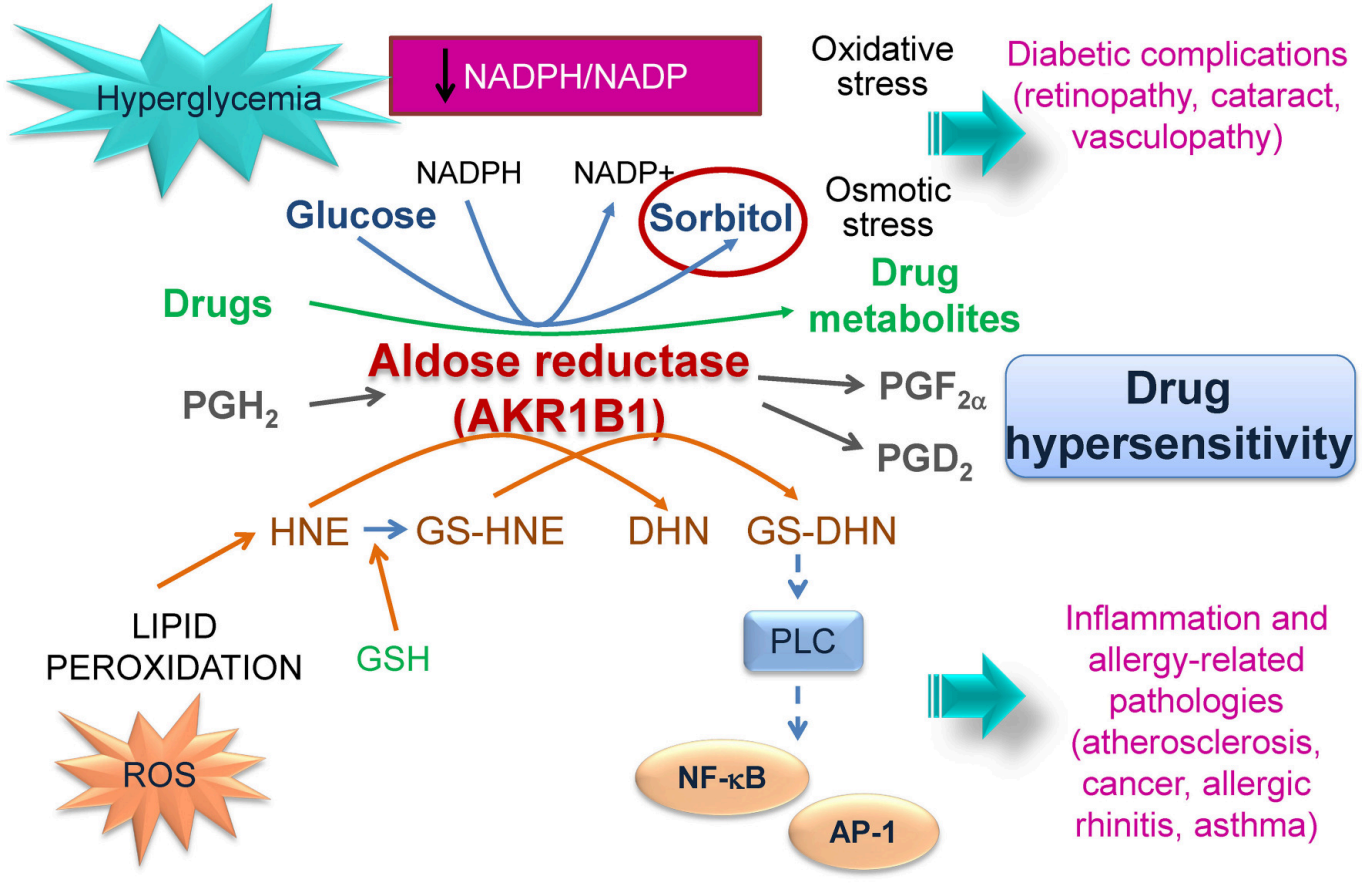
**Involvement of AKR1B1 in pathophysiology**. AKR1B1 catalyzes the first step in the polyol pathway transforming glucose into sorbitol using NADPH as cofactor. Under hyperglycemic conditions increased sorbitol and NADPH consumption lead to osmotic and oxidative stress, respectively, that can contribute to diabetic complications. AKR1B1 metabolizes drugs leading to inactivation and chemoresistance and/or to the generation of toxic metabolites. In addition, AKR1B1 can metabolize PGH_2_ yielding PGF_2α_, which may regulate PGE_2_ production. Transformation of reactive aldehydes or their GSH-conjugates by AKR1B1 can generate species that perpetuate inflammation and may be involved in allergic responses. The interactions of AKR1B1 with drug metabolism, oxidative stress, inflammation an allergic reactions support its consideration in studies of drug hypersensitivity.

Nevertheless, AKR1B1 also has a negative side since it can promote tumor chemoresistance and contribute to the perpetuation of inflammation and to the development of secondary diabetic complications (Figure 2).

### Interactions of AKR1B1 with oxidative stress

AKR1B1 activity is regulated by oxidative posttranslational modifications. The highly nucleophilic Cys298, located near the active site, can be modified by different reactive species like nitric oxide (NO), HNE, or oxidized glutathione. These modifications may reduce or increase AKR1B1 catalytic activity, depending on the modifying moiety, and reduce its susceptibility to pharmacological inhibitors. Interestingly, NADPH protects Cys298 from modification by these agents (Chandra et al., 1997; Del Corso et al., 1998; Petrash, 2004).

AKR1B1 is a target gene of Nrf-2, the master transcription factor regulating the antioxidant response. Therefore, it is induced by numerous oxidative stimuli and participates in the antioxidant response (Kang et al., 2007; Wang et al., 2012). In consequence, AKR1B1 expression is increased in tissues with elevated oxidative stress, e.g., in alcoholic liver disease or vascular inflammation (Srivastava et al., 2005), where in some cases affords a protective role (Kang et al., 2014). Nevertheless, excessive AKR1B1 activity can lead to NADPH depletion and oxidative stress.

This occurs in diabetes, where AKR1B1 metabolizes excess glucose through the polyol pathway. An increased flux through this pathway can lead to osmotic stress due to the increased formation of sorbitol, as well as to a redox imbalance by the elevated consumption of NADPH (Petrash, 2004; Figure 2). NADPH is a substrate/cofactor for several enzymes involved in the cellular antioxidant defense, including glutathione reductase (GSH regeneration), peroxiredoxins and thioredoxin, as well as for several detoxifying systems (Pollak et al., 2007a). Therefore, depletion of NADPH changes the NADPH/NADP^+^ ratio contributing to oxidative stress and reducing the cellular ability to recover after an oxidative insult (Pollak et al., 2007b; Ying, 2008).

### Interaction of AKR1B1 with drugs

AKR1B1 is an important drug target due to its implication in the development of diabetic complications. Therefore, the search for inhibitors from both synthetic and natural sources has yielded a wide array of compounds that bind and/or inhibit the enzyme, with structural information on their binding arising from molecular modeling or crystallographic studies. AKR enzymes are involved in chemoresistance because they metabolize carbonyl-containing drugs, including naloxone and ketotifen (Endo et al., 2014). The anthracycline antibiotics doxorubicin and daunorubicin pose an important case, since they are among the most effective chemotherapic drugs. However, the reduction of their carbonyl group to their corresponding alcohol, yielding doxorubicinol and daunorubicinol, respectively, reduces their efficacy (Veitch et al., 2009). Overexpression of AKR1B1 inactivates these drugs and leads to resistance of various tumor cells (Plebuch et al., 2007; Heibein et al., 2012). Conversely, AKR1B1 inhibition increases the cytotoxic effects of the anticancer agents doxorubicin and cisplatin in HeLa cervical carcinoma cells (Lee et al., 2002), and the AKR inhibitors PGA_1_ and AD-5467 improve the effectiveness of doxorubicin in lung cancer cells (Díez-Dacal et al., 2011; Díez-Dacal and Pérez-Sala, 2012). Natural variants of certain AKR enzymes have been identified that present a reduced capacity to metabolize daunorubicin and doxorubicin *in vitro* (Bains et al., 2008, 2010). There is little information on the involvement of AKR1B1 metabolites in hypersensitivity reactions. Nevertheless, daunorubicinol has toxic effects *per se* because it induces cardiomyopathy (Minotti et al., 2004).

### Interaction of AKR1B1 with inflammatory mediators

AKR1B1 plays an important role in different inflammatory diseases such as atherosclerosis, sepsis, asthma, uveitis, and colon cancer. AKR1B1 can be induced by proinflammatory stimuli (Bresson et al., 2012). Transcription factors Nuclear factor (NF)-κB and AP-1 activate the AKR1B1 promoter through binding to the osmotic response element (ORE; Iwata et al., 1997; Lee et al., 2005) and the phorbol ester response or AP-1 sites, respectively (Penning and Drury, 2007).

Although AKR1B1 can play a protective role by detoxifying acrolein or HNE, it can also play a positive/amplifying role in inflammation through various mechanisms (Figure 2). In particular, metabolism of HNE or its glutathione conjugate GS-HNE can result in products, such as 1, 4-dihydroxynonene (DHN) and glutathionyl-1,4-dihydroxynonane (GS-DHN), which are still toxic and promote activation of phospholipase C (PLC)-NF-κB cascades perpetuating inflammation (Ramana et al., 2006; Srivastava et al., 2011). Thus, inhibition of AKR1B1 reduced NF-κB-dependent inflammatory markers, and the synthesis of TNF-α stimulated by hyperglycemic conditions, and of inflammatory mediators like NO and PGE_2_ (Ramana and Srivastava, 2010).

Interestingly, AKR1B1 displays PGF2α synthetizing activity through which it can regulate PGE_2_ production (Bresson et al., 2012), thus contributing to the modulation of inflammation. In turn, AKR1B1 can bind several PG, including PGE_1_ and PGE_2_ and their cyclopentenone products, PGA_1_ and PGA_2_, which results in inhibition of the enzyme (Díez-Dacal et al., 2016). However, whereas binding and inhibition by PGE appear to be fully reversible, cyPG form a Michael adduct that seems irreversible under certain conditions. Nevertheless, concentrations of GSH in the cellular range (millimolar) elicit a retro-Michael reaction, a fact that contributes to explain the more intense modification and inhibition of some AKRs detected in GSH-depleted cells (Díez-Dacal et al., 2011).

### AKR1B1 in allergic reactions

Early reports linking AKR1B1 to hypersensitivity provided fragmented pieces of evidence. The AKR1B1 inhibitor sorbinil, not currently used in clinical practice, elicited severe adverse effects, including hypersensitivity attributed to protein adducts produced by sorbinil metabolites (Maggs and Park, 1988). Interestingly, lodoxamide tromethamine, and several anti-allergy drugs, inhibit AKR1B1 (White, 1981), providing additional possibilities of interaction with the hypersensitivity response.

Recent studies using pharmacological or genetic depletion establish a positive role for AKR1B1 in allergy. In mice, AKR1B1 inhibition reduced airway inflammation, hyperresponsiveness and IgE and Th2-cytokine levels in ovalbumin and ragweed pollen extract-induced asthma (Yadav et al., 2009, 2011a). Furthermore, studies in AKR^−/−^ mice also support a role of AKR1B1 in the pathogenesis of asthma and allergic rhinitis (Yadav et al., 2011b, 2013a). Moreover, the efficacy of AKR1B1 inhibitors in mouse models supports their use to treat these allergic conditions (Yadav et al., 2011b, 2013a). In mice sensitized with ovalbumin, AKR1B1 inhibition with fidarestat prevented the airway remodeling observed in chronic asthma by blocking the tumor growth factor β (TGFβ), phosphatidylinositol-4,5-bisphosphate 3-kinase (PI3K)/Protein kinase B (PKB/AKT)/Glycogen synthase kinase-3 beta (GSK3B) axis (Yadav et al., 2013b).

The mechanisms linking AKR1B1 with allergy are not fully understood. Nevertheless, it could be hypothesized that it provides coactivators of the allergic response through its contribution to oxidative stress or to the generation of proinflammatory mediators, like aldehyde conjugates.

In contrast to the numerous studies on GSTP1-1 polymorphisms in allergic patients, most genetic studies on AKR1B1 have been directed to explore its association with the development of diabetic implications (Demaine, 2003), and very little information exists on the impact of AKR1B1 variants on drug metabolism or hypersensitivity reactions. Nevertheless, given the fact that an increased glucose flux through the polyol pathway leads to redox imbalance, it would be interesting to assess the involvement of AKR1B1 variants in oxidative stress. In addition, the recent evidences on the involvement of AKR1B1 in allergy grant its study in association with these processes.

In summary, AKR and GST enzymes are emerging as important regulators of the balance of inflammatory mediators. This, together with their association with allergic processes and their ability to metabolize and be covalently modified by drugs makes them attractive candidates to explore their involvement not only in allergy in general but in drug hypersensitivity.

## Author contributions

FS contributed to manuscript writing, figure preparation and experimental work. BD contributed to manuscript writing and figure preparation. EG contributed to manuscript writing. JA contributed to manuscript writing. MP contributed to manuscript writing. DP coordinated and wrote the manuscript and prepared figures.

## Funding

This work has been supported by grants SAF2012-36519 from MINECO and SAF-2015-68590-R from MINECO/FEDER and ISCIII RETIC RIRAAF RD12/0013/0008 to DP, and RD12/0013/0002 to JA.

### Conflict of interest statement

The authors declare that the research was conducted in the absence of any commercial or financial relationships that could be construed as a potential conflict of interest.
